# Research progress of mechanisms of fat necrosis after autologous fat grafting: A review

**DOI:** 10.1097/MD.0000000000033220

**Published:** 2023-03-10

**Authors:** Shenzhen Gao, Baixue Lu, Rong Zhou, Weicheng Gao

**Affiliations:** a Department of Plastic and Cosmetic Surgery, The Affiliated Friendship Plastic Surgery Hospital of Nanjing Medical University, Nanjing, China.

**Keywords:** browning, fat grafting, fat necrosis, fibrosis

## Abstract

Currently, autologous fat grafting is the common surgery employed in the department of plastic and cosmetic surgery. Complications after fat grafting (such as fat necrosis, calcification, and fat embolism) are the difficulties and hotspots of the current research. Fat necrosis is one of the most common complications after fat grafting, which directly affects the survival rate and surgical effect. In recent years, researchers in various countries have achieved great results on the mechanism of fat necrosis through further clinical and basic studies. We summarize recent research progress on fat necrosis in order to provide theoretical basis for diminishing it.

## 1. Introduction

Autologous adipose provides multiple benefits (rich sources, easy harvest, soft texture, flexibility, and low immunogenicity) making it an ideal substitute filler for reconstructive surgery. Although autologous fat grafting become increasingly popular, it creates adverse outcomes including fat necrosis, low survival rate, and fat embolism, which plague surgeons and patients. Fat necrosis is a sterile inflammatory process in the presence of damaging factors, with adipose tissue liquefaction, fibrosis, and calcification.^[1,2]^

As the mechanism of fat necrosis is complex and not very clear, how to reduce it has become a hot and difficult point in the field of fat grafting in recent years. In this review, the science and theory behind fat necrosis are summarized.

## 2. History

Fat necrosis was first reported in the traumatic breast by Lee and Adair,^[3]^ which manifested as palpable hard nodules similar to breast tumors. In 1930, Cookson^[4]^ observed that the nodules incorporated a body of giant cells filled with foam-like cytoplasm and foreign-body large cells, surrounded by fibroblasts. In 1947, Adair and Munzer^[5]^ considered that fat necrosis was produced by a benign aseptic inflammation, acting as various forms of liquefied necrosis, cyst, and calcification by the analysis of the lump formed after breast reconstruction, containing substantial necrotic fat. There were lots of literature regarding fat necrosis after trauma, lumpectomy, implant removal, and so forth. However, fat necrosis associated with autologous was reported until Bircoll^[6]^ got an excellent result over breast augmentation with autologous liposuction in 1987. In 1990, Vizcaino and Montilla^[7]^ touched multiple smooth, firm, and round nodules, with a X-ray examination showing cystic masses by injection of autologous nonvascularized fat in the breast. The histological analysis of masses revealed cystic contents comprised of necrotic fat and fibrous tracts without calcification. Maillard^[8]^ achieved similar outcomes with Vizcaino and the cystic wall with slight calcification. Har-Shai et al^[9]^ detected the liponecrotic cyst by injecting autologous fat obtained by liposuction into the cheek and indicated that leaking oil from adipocyte modulates the inflammatory and granulomatous response to fat necrosis and predisposes to cystic formation. In 2009, the American Society of Plastic Surgeons revised the assessment concerning the current applications and safety of autologous fat grafts and mentioned that the main adverse event was related to fat necrosis, as was perceived to interfere with breast cancer screening and result in graft loss.^[10]^ Since then, significant attention to fat necrosis related to autologous fat transfer has been well described.

## 3. Pathogenesis

Clinically, it is hard to distinguish calcification between fat necrosis and breast cancer on imaging; thus, identification of distinct pathological features of fat necrosis helps clarify its diagnosis and assess the severity degree and stages.

Fat necrosis is the product created by sterile inflammation, as the result of aseptic saponification of lipases in blood and tissues.^[2,11,12]^ Histopathological examination is the gold standard for the diagnosis of fat necrosis. Histologically, numerous fibroblasts, coenocytic giant cells, and lipid-filled macrophages clustered around varying vesicles merged by ruptured fat cells in the grafting area.^[13]^ Berg et al^[14]^ subdivided fat necrosis into 3 stages according to pathological manifestations: early stage, where fragments of fat cells were visible; middle stage, where red blood cells and phagocytes infiltrated; late stage, where multinucleate giant cells, hemosiderin, and calcification were noted. It was noticed that aggregated phagocytes around the necrotic region were mostly M2 macrophages through autologous fat grafting in mice.^[15]^ In a similar experiment, Kato et al^[16]^ revealed that all of the adipocytes and adipose-derived stem cells were dead, with the occupation of extracellular matrix, oil cysts, as well as calcification. The cyst wall consisted of both inner and outer layers in the oil cyst with a long history of autologous fat injection into breast augmentation. From the comparison between short and long case history, calcification was limited to the innermost fibrous area in the former and was stronger on both internal and external layers in the latter.^[17]^ Under the transmission electron microscope, grafts were characterized by cellular swelling, mitochondrial cristae loss, chromatin condensation, and broken cytomembrane.^[18]^ The above indicates that fat necrosis may present diverse appearances in different phases.

## 4. Associated mechanisms

Fat necrosis after autologous fat grafting is the consequence of multiple inflammatory factors and tissue cells in response to specific stimuli. Precious studies indicated that the pathogenesis of fat necrosis includes ischemia and hypoxia, white fat browning, and fibrosis.

### 4.1. Hypoxia

The survival of autologous fat grafts depended on the reestablishment of blood circulation.^[11,19]^ Following the implantation into the recipient area, in the early stage (generally within 4 weeks), tissue fluid provided nutrients, and neovascularization allowed for graft survival at a later stage. Without adequate nutrients meeting adipose tissue requirements, portions underwent liquefied death. Fat absorption following revascularization mediated apoptosis without the inflammation associated with membrane rupture or lipid droplet leakage.^[20,21]^ Accordingly, in the context of hypoxia fat necrosis provoked graft volume depression rather than apoptosis. More oxygen was needed in adipose tissue than in others because of higher oxygen tension in subcutis. Within the same volume, the bloodstream was richer in adipose than skeletal muscle; hence, fatty tissue was susceptible to ischemic necrosis in the presence of dissection of vessels.^[11,22]^ Inflammatory reflection induced release agents (tumor necrosis factor-α [TNF-α], interleukin [IL]-6, etc.) to defend against microenvironmental changes in the setting of hypoxia, but excess agents could destroy normal adipocytes. The less inflammatory response observed in fat flap with vascular anastomosis could reduce the fat necrosis morbidity for a comparison between the autologous free fat and fat flap grafts with vascular anastomosis in mice by Oashi et al^[23]^ and Mashiko and Yoshimura^[20]^ observed through autologous fat grafting experiments in mice that the majority of adipocytes started to die by the time the oxygen tension was below half initially; while oxygen continued to decrease, endothelial cells and hemogenic cells suffered an injury. In the condition of subcutaneous oxygen tension beneath the threshold of 30 to 35 mm Hg (normal is 50–60 mm Hg), there was irreversible damage to adipocytes; nevertheless, dying adipocytes could be reversed to be healthy in case of higher oxygen tension (about 60% or more of normal pressure).^[15]^ Hypoxia-inducible factor-1α and fibroblast growth factor-2 were up-regulated in severe hypoxia, involving the occurrence of fat necrosis. During the trial of the mice model lacking oxygen, Eto et al^[24]^ demonstrated that adipose-derived stem cells, precursor cells, and stromal cells worked together to diminish the cyst structure. Consequently, to avoid negative outcomes after the autologous fat graft, restoration of blood supply for implantation is required.

### 4.2. Browning

Aside from 2 classic types of fat tissue, white adipose tissue (WAT) for storing the energy and brown adipose tissue, there was a novel fat tissue known as “beige” or “brite” adipose tissue, which localized at the inguinal, axillary, and subcutaneous tissues as WAT did, with similar morbidity and function to brown adipose tissue. Beige adipocytes harbored multilocular lipid droplets and abundant mitochondria with high expression of uncoupling protein-1, prone to release high heat. Under the stimulation of cold or drugs, the quantity and activity of beige fat increased significantly in the subcutaneous white fat areas, which was known as the “ browning of WAT.”^[25–27]^ The origin of beige fat during browning was still debated: part of scholars believed that it derived from adipose precursor cell differentiation, and others argued that it was converted by white adipocytes. Browning of WAT could be induced after the procedure of autologous fat transfer in mice by Hoppela et al^[28]^ When female abdominal fat was implanted to the back of nude mice by fat aspiration, graft browning was noted mainly in the late stage, which was recognized as an adoptive and reversible process. After removal of stimuli and inducible agents, the beige fat could transform into white fat; whereas factors were not eliminated, beige fat could remain for a long time and gradually necrotize.^[29]^ Browning fat could be found in fat necrosis, not in well-survived adipose tissue while human adipose tissue was transferred into the back of nude mice by Liu et al.^[30]^ They discussed that the degree of browning is closely related to the illness of the local environment and fat necrosis, as was not a natural process after autologous fat grafting. The authors also found that M1 macrophages secrete IL-6 after fat grafting, promoting the polarization of M2 macrophages, which in turn induced the browning of white fat, but there was no evidence elucidating the exact pathway of it.^[30]^ It has been shown that beige fat was characterized by strong metabolism and high mitochondrial protein; compared to white fat, beige fat was more vulnerable to topical pro-apoptotic factors, inflammatory response, and hypoxia, which made it more susceptible to necrosis.^[26,27]^ Beige fat metabolizes vigorously and consumes more oxygen, further worsening the local hypoxia environment and leading to a vicious cycle. Browning of WAT could be back towards the initial state following the withdrawal of triggers; therefore, by deepening the basic science of browning, the burgeoning surgeon may diminish the occurrence of undesirable fat necrosis.

### 4.3. Fibrosis

Fibrosis is a process of tissue repair engaged by fibrous connective tissue in response to the loss of cells and tissue, which is marked by excessive deposition of extracellular matrix (ECM).^[31]^ In fat necrosis following fat grafting, necrotic fat was retained at the graft region for a long while, surrounded by a fibrous cyst wall; a persistent cyst wall would result in chronic inflammation, but its thickness would block the entrance of inflammatory cells and factors and absorption of oil.^[32,33]^ As such, persistence of oil cysts would induce long-term chronic inflammation with progressive calcification and fibrosis. After autologous fat grafting in the mice model, first of all, neutrophils arrived at the grafts to swallow detrimental elements and secret inflammatory mediators within 24 hours, which was involved in the fibrous process by metalloproteinase-9 decomposing ECM.^[17]^ Liu et al^[31]^ found that long-term oil cysts had a larger size and stronger fibrosis with a lack of neutrophils; thus, the authors considered that upregulation of neutrophils could reduce the fibrotic effect, but the excess would damage other healthy tissues.

Macrophages are the critical cells associated with postnecrotic fibrosis. Within the area of fat necrosis, ruptured adipocytes caused a local inflammatory response, with neutrophils acting as front-line inflammatory cells that gradually subsided after 3 days; at this time, large amounts of macrophages accumulated to engulf the necrotic cells and removed cellular debris and contents. Kato et al^[16]^ studied autologous fat grafting in mice and observed that necrotic adipose tissue was initially infiltrated by M1 macrophages, which released TNF-α, IL-6, IL-2, and other inflammatory factors to eliminate necrotic stuff; when the necrotic substance was so much that M1 macrophages could not absorb all of it, M2 macrophages progressively substituted M1 macrophages as the dominant cells, partially forming fibrous capsules to encapsulate necrotic tissue and the others encapsulating M1 macrophages containing lipid droplets to a crown-like structure. Tanaka et al^[34]^ suggested that M2 macrophages could express C-type agglutinin, which stimulated myofibroblasts to secrete collagen and promoted the production of coronal-like structures.

By transplanting human abdominal adipose tissue to the back of nude mice, Cai et al^[35]^ revealed that M2 macrophages in the fat necrosis area could highly express transforming growth factor-β, contributing to fibroblast proliferation and differentiation, and the synthesis of multiple ECM proteins by adipose precursor cells. In addition, M2 macrophages could also secrete collagen by themselves, and in the presence of a lack of M2 macrophages, a decrease in type I collagen, type VI collagen, capsule wall thickness and fibrosis was evident. Abnormality of accumulated lipid allowed for necrosis of macrophage foam cells across the RIPK3/MLKL signaling pathway, which was produced by the fact that macrophages phagocytosed broken adipocytes, and the release of inflammatory factors and chemokines (TNF-α, IL-1α, IL-6, and monocyte chemotactic protein-1), facilitating the secretion of enormous collagen by fibroblasts.^[36]^

The size and quantity of fat determined the fibrous level of fat necrosis. Fat droplets less than 1mm in diameter can be completely absorbed within a few weeks, while those with a diameter of more than 3 mm are more potential to die. After transplanting fat aspirated from the human abdomen to the back of nude mice, where more oil was transplanted, the more obvious was the liquefied necrosis, macrophage inflammatory infiltration, and fibrosis of the grafts.^[37]^ Fibrosis manifests histopathologically as oil cysts and calcification, and clinically as long-standing palpable hard nodules, local skin retraction or thickening as well as inflammatory reactions such as redness, swelling, and heat pain in patients, giving rise to repeated postoperative visits. Additional studies are necessary to substantiate these findings and the utility of each method, which helps to provide strategies for precaution and treatment of fat necrosis, and new levels of improved patient satisfaction with superb aesthetic results will follow.

## 5. Conclusion

Autologous fat grafting has been practiced widely today, it is vital for plastic surgeons to be familiar with its negative outcomes, with special care for fat necrosis, which determined the survival rate of transplanted fat. Moreover, fat necrosis causes calcification, cysts, and inflammatory reactions that can interfere with disease diagnosis and treatment. Currently, studies on fat necrosis mainly focused on the histopathology of fat necrosis masses, fat grafting in mice, etc. The mechanisms include the establishment of blood transport, browning of white fat, fibrosis after fat necrosis, etc. There is little evidence focusing on the molecular mechanisms of fat necrosis. A comprehensive summary of the science and theory of fat necrosis behind autologous adipocyte grafting, as well as its rich history is described. It is our belief that the review can set a path for strategies for the treatment of fat necrosis.

## Author contributions

**Conceptualization:** Shenzhen Gao, Weicheng Gao.

**Writing – original draft:** Shenzhen Gao, Baixue Lu.

**Writing – review & editing:** Rong Zhou, Weicheng Gao.
